# The mediating role of perceived overqualification in the relationship between emotional labor and mindfulness among nurses in China

**DOI:** 10.3389/fpubh.2025.1519192

**Published:** 2025-03-17

**Authors:** Yangyang Han, Aihua Su, Yi Xuli, Yueming Lv, Fujie Jing

**Affiliations:** ^1^School of Acupuncture-Tuina, Shandong University of Traditional Chinese Medicine, Jinan, China; ^2^School of Nursing, Shandong First Medical University and Shandong Academy of Medical Sciences, Jinan, China

**Keywords:** nurse, mindfulness, perceived overqualification, mediation, emotional labor

## Abstract

**Background:**

Perceived overqualification has attracted considerable attention from labor economists and managers, who perceive it as a risk factor affecting workforce stability. Mindfulness is closely associated with emotional labor, however, the potentiatl mechanisms underlying the relationship between mindfulness and emotional labor remain ambiguous. This study seeks to investigate the mediating role of perceived overqualification in the relationship between emotional labor and mindfulness among clinical nurses.

**Methods:**

A total of 354 clinical nurses were assessed using the Mindful Attention Awareness Scale, the Perceived Overqualification Scale, and the Emotional Labor Scale. The SPSS version 27.0 statistical analysis software was employed for the basic organization and analysis of the survey data. A structural equation model was used to assess the mediating role of perceived overqualification between mindfulness and the emotional labor of clinical nurses.

**Results:**

The study indicated that the level of mindfulness and emotional labor among clinical nurses was moderate to high, whereas perceived overqualification was moderate. A significant negative correlation was observed between perceived overqualification and mindfulness (r = −0.270, *P* < 0.001). In contrast, mindfulness was significantly positive correlated with deep acting (r = 0.110, *P* < 0.05) and significantly negatively correlated with surface acting and emotional expression requirements (r = −0.294, r = −0.278, *P* < 0.001). Furthermore, perceived overqualification acted as a mediator between mindfulness and surface acting as well as between mindfulness and emotional expression requirements, with mediating effect sizes of 20% and 12.5%, respectively.

**Conclusion and recommendation:**

By promoting mindfulness among clinical nurses while reducing their perceived overqualification may enhance their emotional labor capabilities, thereby fostering positive outcomes for their physical and mental health, and contributing to the advancement of high-quality nursing services.

## Introduction

In the realm of mental and emotional wellbeing, the concept of mindfulness holds considerable significance.

Originating from the Buddhist traditions, mindfulness essentially encapsulates three key elements: awareness, attention, and remembering. However, despite the growing ubiquity and applications of mindfulness, its definition remains somewhat fluid and open to interpretation. Scholars from diverse academic backgrounds have attributed varying meanings to this concept. For instance, Baer (1) views mindfulness as a method of concentration, while Kabat-Zinn (2), and Teasdale et al. (3) define it more broadly as a purposeful awareness of both internal and external experiences in the present moment. Bishop et al. (4) and Tusaie and Edds (5) interpret mindfulness as a mental process, suggesting it encompasses cognitive and psychological elements. Furthermore, Brown and Ryan (6) and Davidson et al. (7) have conceptualized mindfulness as a dual entity, representing both a trait and a state. As traits, individuals may exhibit varying tendencies toward mindfulness in their daily routines, which in turn influences their state of mind and attention toward external experiences. The most widely accepted definition of mindfulness, however, seems to align more with Kabat-Zinn's (2) original proposal, describing it as a purposeful, non-evaluative awareness of the momentary experience of the present moment. This understanding positions mindfulness as a deliberate and active engagement with one's thoughts and surroundings.

The effectiveness of mindfulness practice has been corroborated by a plethora of studies. Biologically, mindfulness interventions have been linked to a range of physiological changes. For instance, participants often exhibit decreased cortisol levels, indicating reduced stress response (8, 9). Similarly, improved immune function (10) and lower blood pressure readings (11, 12) have also been observed. Furthermore, mindfulness practice has been clinically demonstrated to provide relief from chronic pain (13–15), particularly in cases of chronic back pain (16). At the psychological level, mindfulness has proven to be beneficial in multiple ways. Stress reduction is perhaps the most common psychological benefit associated with mindfulness (17). Regular mindfulness practice can help individuals cope better with stress, leading to improved mental health and overall wellbeing. Moreover, mindfulness has been shown to be effective in managing symptoms of anxiety and depression. The practice seems to enhance mood stability and overall psychological wellbeing. The benefits of mindfulness are not merely theoretical or anecdotal. Solid evidence from various scientific quarters supports the effectiveness of mindfulness interventions. Studies employing neuroscientific methods have provided tangible evidence of changes in the brain resulting from mindfulness meditation. These changes are both structural and functional, reducing the amygdala's response to emotional stimuli and increasing gray matter density (18, 19) as well as cortical thickness in the insular and prefrontal cortex (20). Such changes are beneficial for mental health, as they indicate better regulation of emotions. The high-stress occupation of being a nurse has made them a primary target for mindfulness researchers. Numerous studies have demonstrated that mindfulness interventions can help alleviate or even eliminate physical and mental symptoms often associated with prolonged stress. These include improved sleep quality, reduced symptoms of job burnout (21–24), and enhanced life satisfaction, all of which contribute to better stress management and reduced burnout (25, 26). Additionally, mindfulness practice has been linked to improved memory function (27), highlighting one of the many cognitive benefits. The results of mindfulness interventions are not limited to a single culture or setting. A Malaysian scholar, Lan et al. (28), employed a quasi-experimental study design, revealing significant reductions in participants' levels of perceived stress, anxiety, and depression. Similarly, Gauthier et al. (29) demonstrated a notable decrease in stress levels among pediatric ICU nurses who participated in a brief, 5-min mindfulness-guided session before their morning and late shifts. Spanish scholar Gozalo et al. (30) utilized a mobile application to conduct mindfulness training over an 8-week period with 32 ICU medical staff, resulting in improvements in the emotional exhaustion dimension of burnout. This array of evidence, from diverse geographical and cultural contexts, underscores the widespread applicability and effectiveness of mindfulness interventions.

In 1983, sociologist Hochschild (31) was the first to introduce and propose the concept of emotional labor, while she was deeply examining the ways in which the service attitudes of airline employees influenced their work. As an extension of her study on the impact of service attitudes on work, Hochschild defined emotional labor as a process in which individuals are required to manage and control both their internal emotions and external expressions to align with the expression requirements set by their respective organizations. This idea marked a significant shift in understanding the emotional aspect of work, as it moved beyond the mere discussion of feelings and entered the realm of active emotion management. In 1992, Smith et al. (32), a nursing scholar, formally introduced the concept of emotional labor into the nursing industry, characterizing it as an essential requirement for nurses to adjust their feelings and emotions according to varying situations and contexts encountered in their line of work. She emphasized that this adjustment was crucial for fostering and maintaining a positive rapport between caregivers and patients, thereby ensuring that the care provided was authentic and effective. Then, in 2000, Grandey (33) conducted extensive research, drawing upon the theory of emotion regulation to establish that emotional labor encompasses a series of psychological adjustment activities undertaken by individuals to align with the rules of emotional expression expected by their respective organizations. He highlighted the dynamic nature of this process, which involves the application of emotion regulation strategies to manage and navigate the emotional expression required by one's job. Building upon the foundations laid by Hochschild, Pam, and Grandey, Difendorff (34) in 2003 utilized a self-regulated control model and psychological cybernetics to further our understanding of emotional labor, positing that it is indeed a dynamic process that requires the application of emotion regulation strategies to manage and navigate the emotional expression demands of one's job.

The implications of emotional labor extend beyond the realm of sociological and psychological studies. It is particularly prevalent in industries that are service-oriented, such as in the case of hotel employees (35), teachers (36), and social workers (37). Within the nursing profession (38), emotional labor takes on an evegreater significance given the daily encounters and management of negative emotions like anxiety and fear from patients and their families. The emotional labor burden for nurses is substantial, with hospice nurses (39) and oncology nurses (40) operating at an upper middle level of emotional labor, while nurses in Korea also experience a medium-to-high level of emotional labor (41). Wo's research (42) demonstrated that emotional labor has a direct impact on the job satisfaction and performance of clinical nurses. Subsequently, a growing body of research is aimed at understanding the factors that influence emotional labor. This line of inquiry primarily examines the influence of personal characteristics, work environments, and psychological traits. Gulsen's study (43) found that women, compared to men, tend to exhibit better emotional regulation, while men, on the other hand, may suppress their true emotions, which could exacerbate the emotional labor demands of their work. Furthermore, Delgado et al. (44) and Al-Hamdan et al.'s research (45) indicated that nurses with high emotional intelligence are more adept at regulating their emotions, thus enabling them to cope more effectively with emotional demands, and as a result, tend to exhibit lower levels of emotional labor.

Perceived overqualification is a construct that emerges from the concept of educational overqualification (46), a term used to describe an individual's belief that their educational background and abilities surpass the requirements of their current job role (47). This idea is further developed by Maynard, who integrates these sub-concepts into a higher-level construct. This higher-level construct is defined as the degree to which an individual feels their qualifications exceed the needs of their position. This construct is crucial as it signifies a subjective perception, which is predominantly studied within the disciplines of psychology and organizational behavior. Objective measures of overqualification often tend to overlook the nuances of individual differences and subjective experiences. As a consequence, people's attitudes and behaviors are frequently influenced more by their subjective evaluations of objective circumstances than the circumstances themselves (48).

The phenomenon of overqualification has garnered substantial attention from labor economists and managers. These professionals view it as a risk factor that influences workforce stability (49). Overqualification is not merely an academic concept; it has real-world implications for various professionals, including nurses. The phenomenon can adversely impact nurses' psychological and cognitive wellbeing. It can lead to a range of negative emotions, diminished work performance, lower job satisfaction, reduced organizational identity, and decreased overall happiness at work (50). Moreover, nurses who experience overqualification are more susceptible to professional burnout (51). In examining the relationship between nurses' overqualification and emotional labor, emotional labor is considered an antecedent variable, whereas the perception of overqualification is regarded as an outcome variable. Research indicates that overqualification may exacerbate the depletion of employees' psychological resources, compelling them to adopt surface-acting strategies in their emotional labor. This may result in lower job satisfaction and diminished psychological wellbeing (52, 53). However, some studies suggest that individuals with lower levels of overqualification can effectively and proactively manage their emotions. These employees often possess robust psychological capital, which can help ameliorate the negative cognitive effects linked with emotional labor.

Correlations between emotional labor, mindfulness, and perceived overqualification have been established in various studies. However, the relationship between mindfulness and perceived overqualification, particularly how the latter develops and whether it can be influenced by mindfulness, remains an area of research that requires further investigation. Moreover, while it is known that correlations exist among mindfulness levels, perceived overqualification, and emotional labor, these specific correlations, especially among clinical nurses, have not been previously reported in the literature. Thus, this study aimed to investigate the correlations between nurses' mindfulness levels, perceived overqualification, and emotional labor, as well as to explore the mediating role of perceived overqualification in the relationship between mindfulness and emotional labor among clinical nurses. The findings of this study hold great significance for nursing management as they can contribute to a theoretical foundation for enhancing clinical nurses emotional labor and implementing targeted psychological interventions. By encouraging nurses to elevate their mindfulness levels and effectively manage feelings of perceived overqualification, leverage the positive effects of emotional labor, safeguard their physical and mental health, and ultimately improve their medical experiences. In summary, the purpose of this study is to explore the mediating role of perceived overqualification in the effect of emotional labor and mindfulness among clinical nurses, and the results of this study have important implications for nursing management in providing a theoretical foundation for enhancing clinical nurses emotional labor and implementing targeted psychological interventions.

## Methods

### Design

This cross-sectional study was conducted at three Triple A Hospitals in Jinan, Shandong Province, China, with convenience sampling.

### Participants

Nurses were invited to participate in an online questionnaire survey using WeChat and QQ social software. The inclusion criteria's were as follows: (1) participants must be licensed and continuously registered nurses and (2) they must provide consent to participate in this study. The sample size was calculated prior to data collection using the G^*^power software version 3.1. The researcher estimated the sample size based on a desired power of 0.80, significance level (α) of 0.05, and medium effect size of 0.5. Initially, 370 nurses from three hospitals participated in this study. After excluding questionnaires with excessively fast response times, strong regularity in answers, and a high number of missing values, the researchers obtained 354 valid questionnaires, resulting in an effective response rate of 95.68%.

### Measurements

#### Mindful attention awareness scale

The scale was developed and validated by Brown and Ryan (6), and subsequently translated and revised into Chinese by Chen et al. (54). The MAAS utilized in this study consists of 16 items rated on a 6-point Likert scale. Higher scores indicate greater levels of attention and awareness of the present moment in daily life, reflecting elevated levels of mindfulness. The total score categorizes mindfulness into three distinct levels: scores ranging from 66 to 90 indicate high mindfulness, scores from 41 to 65 represent medium mindfulness, and scores below 40 indicate low mindfulness. The Cronbach's alpha coefficient of this scale was 0.890.

#### Scale of perceived overqualification

The SPOQ was developed by Chun (55) and subsequently translated into Chinese and revised by Pinkawa and Dörfel (56), based on a framework that holistically measures knowledge, education, and culture within the Chinese context. The scale consists of nine items and is scored using a 5-point Likert scale, ranging from 1 (not at all conforming) to 5 (fully conforming). This demonstrated an internal consistency coefficient of 0.850. The total score ranged from 9 to 45, with higher scores indicating a greater degree of self-perceived overqualification in the workplace. The Cronbach's alpha coefficient of this scale was 0.845.

#### Nurse emotional labor scale

The NELS was developed by Botha (57) and subsequently translated into Chinese, and revised by Bögels et al. (58). The scale comprises three dimensions with a total of 14 items: 7 items for surface acting, 3 items for deep acting, and 4 items for emotional expression. The scale operates on a 6-point Likert scale, ranging from “strongly disagree” to “strongly agree,” with scores ranging from 1 to 6. Cronbach's alpha coefficients for the three subscales and the overall scale were 0.711, 0.826, 0.872, and 0.811.

### Data analysis

The SPSS version 27.0 statistical analysis software was employed for the basic organization and analysis of the survey data. There were only a few missing values in the general demographic information that were replaced with the mean value. Descriptive statistics were performed for the general demographic data, with frequency and percentage (*n*, %) used for count data, and mean ± standard deviation used for measurement data. Pearson's correlations were used to examine the correlations between the variables. A structural equation model was used to assess the mediating role of perceived overqualification between mindfulness and the emotional labor of clinical nurses.

### Ethical considerations

All procedures in this study during the survey process adhered to the ethical standards for human experimentation established by the Ethics Committee and the revised 1975 Declaration of Helsinki. This study was approved by the Human Subjects Ethics Subcommittee of Shandong First Medical University (registration number: R202306170189). The participants signed an online informed consent form, acknowledging their understanding of the purpose and significance of the study. Verbal consent was obtained from all participants prior to administering the questionnaire. This study was conducted using an anonymous survey, ensuring that no specific identifying information of the participants (e.g., IP address and name) was collected, thereby protecting the personal information of each participant.

## Results

### Sample demographics

This study involved 354 nurses, including 59 (16.70%) male nurses and 295 (83.30%) female nurses. The average age of the participants was 25.24 ± 1.05 years, and the educational attainment was primarily represented by undergraduates, comprising 324 (91.50%) individuals. The number of night shifts performed by nurses per month was predominantly < 6, with 192 (54.2%) nurses working fewer shifts and 162 (45.8%) nurses working more than six shifts. Additionally, marital status was largely dominated by married individuals (64.10%).

### Correlation analysis

Perceived overqualification was significantly negatively correlated with the level of mindfulness and significantly positively correlated with Emotional labor, providing support for hypothesis1. (1) Perceived overqualification was significantly negatively correlated with mindfulness (r = −0.270, *p* < 0.001) and significantly positively correlated with emotional labor, surface acting and emotional expression (r = 0.254, r = 0.207, r = 0.266, *p* < 0.001). (2) Mindfulness was significantly positively correlated with deep acting (r = 0.110, *p* < 0.05) and significantly negatively correlated with emotional labor surface acting and emotional expression (r = −0.236, r = −0.294, r = −0.278, *p* < 0.001) (Table 1).

**Table 1:** The analysis of Pearson's correlation revealed correlations among the measured variables.

	**1**	**2**	**3**	**4**	**5**	**6**
1 Mindfulness	1					
2 Perceived overqualification	−0.270^***^	1				
3 Emotional labor	−0.236^***^	0.254^***^	1			
4 Deep acting	0.110^*^	−0.002	/	1		
5 Surface acting	−0.294^**^	0.207^***^	/	−0.021	1	
6 Emotional expression	−0.278^***^	0.266^***^	/	0.133^*^	0.572^***^	1

^*^p < 0.05, ^**^p < 0.01, and ^***^p < 0.001.

### Mediation effect analysis

To validate the mediating role of perceived overqualification between the level of mindfulness and emotional labor, as well as to explore the intrinsic mechanisms by which mindfulness influences emotional labor, further mediation analyses involving overqualification, mindfulness levels, and the three dimensions of emotional labor were conducted.

The results indicated that mindfulness had a direct effect on emotional expression, while serving as a mediating factor through the variable of perceived overqualification. Specifically, the direct (−0.108) and mediating (−0.027) effects accounted for 80% and 20% of the total effect (−0.135), respectively (Table 2). The level of mindfulness influenced surface acting in emotional labor both directly and as a mediating factor through the variable of perceived overqualification, with the direct effect (−0.154) and mediating effect (−0.022) comprising 87.50% and 12.50% of the total effect (−0.176), respectively (Table 3). Furthermore, the level of mindfulness influenced deep acting in emotional labor both directly and as a mediating factor through the variable of perceived overqualification, with the direct effect (0.106) and mediating effect (0.058) comprising 78.50% and 21.50% of the total effect (0.164), respectively (Table 4).

**Table 2:** The mediating effects of perceived overqualification between mindfulness and emotional expression.

	**Efficacy value**	**SE**	**LLCT**	**ULCT**	**Quantity of effect**
Total effect	−0.135	0.025	−0.184	−0.086	
Direct effects	−0.108	0.025	−0.158	−0.058	80%
Mediating effect	−0.027	0.011	−0.107	−0.018	20%

SE, standard error; LLCT, lower limit of the confidence interval; ULCT, upper limit of the confidence interval.

**Table 3:** The mediating effects of perceived overqualification between mindfulness and surface acting.

	**Efficacy value**	**SE**	**LLCT**	**ULCT**	**Quantity of effect**
Total effect	−0.176	0.031	−0.237	−0.116	
Direct effects	−0.154	0.032	−0.216	−0.092	87.50%
Mediating effect	−0.022	0.014	−0.054	−0.001	12.50%

**Table 4:** The mediating effects of perceived overqualification between mindfulness and deep acting.

	**Efficacy value**	**SE**	**LLCT**	**ULCT**	**Quantity of effect**
Total effect	0.164	0.027	0.243	0.129	
Direct effects	0.106	0.030	0.209	0.074	78.50%
Mediating effect	0.058	0.017	0.047	0.006	21.50%

## Discussion

This study aims to comprehensively understand the intricate mechanism underlying the psychological impact of perceived overqualification on nurses' emotional labor and mindfulness. Through a series of in-depth analyses, the results of this study demonstrated that perceived overqualification has a significant direct effect on the relationship between emotional labor and mindfulness. Specifically, the study demonstrated a robust negative correlation between perceived overqualification and mindfulness. One potentially underlying explanation for this intriguing finding lies in the concept of mindfulness as a crucial psychological protective factor. Mindfulness encourages an intense focus on the present moment, enabling individuals to better regulate their thoughts and maintain emotional stability (59–61). In contrast, perceived overqualification refers to the self-perception held by nursing professionals that they possess an excessive number of qualifications compared to what is required for their respective roles. Intriguingly, the research findings revealed that higher levels of perceived overqualification are closely correlated with a reduced ability to regulate thoughts and a lower level of mindfulness (62, 63). Recognizing the importance of nurturing nurses' psychological wellbeing, it is crucial to address these issues by providing guidance and support. This could involve regularly encouraging nurses to reflect on their thinking patterns, raising awareness of their perceived overqualification levels, and implementing mindfulness stress reduction training at timely intervals. Furthermore, fostering team discussions to share experiences and strategies related to mindfulness training while coping with perceived overqualification can be beneficial. By reducing perceived overqualification through enhanced mindfulness, nursing professionals can improve their professional identity and strengthen the stability of the nursing team as a whole. This multi-faceted approach can foster a positive work environment that values mindfulness and emotional intelligence, ultimately benefiting both the nurses and the patients they care for.

The study indicated a strong and significant positive correlation between mindfulness and deep acting in emotional labor, signifying that mindfulness enables clinical nurses to engage more fully in their work. In contrast, mindfulness was negatively correlated with surface acting and emotional expression, suggesting that an excessive reliance on surface acting can exacerbate the challenges of nursing work. This is consistent with previous research in the field (64, 65). The study attributes these findings to the fact that a high level of mindfulness enables individuals to be more present, fostering openness to accept and allow experiences as they occur (66). This mental state of mindfulness can protect the psyche and inhibit the emergence of negative emotions, allowing nurses to exhibit a kind and friendly demeanor when interacting with patients. Conversely, surface acting in emotional labor involves a form of “pretend” emotions; excessive reliance on surface acting can intensify the challenges of nursing work, diminishing the quality of psychological care and increasing the likelihood of conflict with patients. Emotional expressions imposed on nurses as part of their emotional labor can be detrimental, particularly for those with low mindfulness. Prolonged exposure to these emotional expression demands may shift emotional labor from deep acting to surface acting (67–69), creating a vicious cycle that undermines nursing care quality and risks fostering rigidity in nurse-patient relationships, ultimately affecting patient care. Nursing managers should prioritize the psychological wellbeing of nurses (70), mitigate the pressures associated with emotional labor (71), and provide mindfulness and emotional labor training (72). This training can help nurses to understand the interplay between the two and explore the positive impact of deep play on nursing. Mindfulness can enhance nurses' focus and emotional labor by increasing their awareness of their own emotions while allowing them to better attend to patients' needs (73, 74). This practice enables nurses to express their emotions more authentically and encourages continuous learning and engagement with mindfulness techniques. Additionally, nurses are encouraged to cultivate positive thinking to improve their emotional labor capabilities, facilitating a gradual transformation from superficial engagement to deeper and more meaningful interactions.

The findings of this research study serve to enrich our understanding of the complex interplay between perceived overqualification, the demands of emotional labor, and the role of mindfulness in shaping an individual's emotional expression and surface acting. The research outcomes, which are in alignment with several prior studies in the field (75–77), indicated a robust positive correlation between the sense of professional overqualification and the requirements for both emotional expression and surface acting. This correlation signifies that as the perception of overqualification intensifies among nursing professionals, their enthusiasm for work tends to wane, leading to a subsequent reduction in the emotional labor they are able to provide. In other words, when the emotional demands of nursing work become perceived as excessive or unwarranted, it can impact negatively on the emotional labor that nursing professionals are able to deliver. As a potential coping mechanism, some nursing professionals may find themselves resorting to what is known as “superficial acting” or the display of emotions for the sake of fulfilling emotional expression requirements (78). This behavior, while understandable, may not address the underlying issue of overqualification and its impact on emotional labor. As such, it becomes crucial for organizational managers to develop and implement strategies that can help to mitigate the perceived overqualification among nursing professionals. One recommended approach is to encourage nurses to engage in reflective practice that can help them to better understand and appraise their professional value. This can involve activities that enhance their skills and foster a greater sense of professional benefit, thereby improving their professional identity (79). Such an approach can also help to create a more positive departmental atmosphere, provide timely positive feedback, acknowledge nurses' performance, and ultimately reignite their enthusiasm for work. Consequently, by fostering a renewed sense of enthusiasm, nursing professionals are better equipped to engage fully in emotional labor, thereby enhancing their capacity to meet the emotional expression and surface acting requirements of their profession.

### Strengths and limitations of the study

This study indicates that mindfulness among clinical nurses can indirectly influence emotional labor through perceived overqualification. Mindfulness can help nurses regulate their emotions and transition from surface acting to deeper emotional engagement, enhancing the quality of care provided to patients. The study also suggests that higher mindfulness may be negatively correlated with perceived overqualification. By fostering mindfulness, nurses can reduce overqualification and surface acting. Managers should consider strategies to enhance nurses' emotional labor capacity, such as stress reduction training, regular professional skills training, and optimizing work practices to prioritize nurses' mental health. These measures can improve mindfulness, reduce perceived overqualification, increase professional identity, and ultimately improve emotional labor capacity, elevating the quality of nursing services and safeguarding nurses' psychological wellbeing.

This study has several limitations. First, the cross-sectional design did not establish causality between these variables. Second, convenience sampling was used to recruit participants from nurses in Shandong Province, China. Therefore, caution should be exercised when extrapolating these findings to other populations, and more controlled and larger-sample studies are needed. Despite these limitations, this study has both theoretical and practical implications.

## Conclusion

There is a correlation among clinical nurses' levels of positive thinking, their sense of overqualification, and emotional labor. Perceived overqualification serves as a partial mediator between clinical nurses' levels of positive thinking and emotional labor. Specifically, nurses' levels of positive thinking can directly influence emotional labor and indirectly affect it through perceived overqualification. Furthermore, perceived overqualification mediates the relationship between positive thinking and emotional labor, particularly in the context of the surface acting required for emotional expression, with contributions of 20% and 12.5%, respectively.

## Data Availability

The datasets presented in this study can be found in online repositories. The names of the repository/repositories and accession number(s) can be found in the article/supplementary material.

## References

[1] Baer RA. Mindfulness training as a clinical intervention: a conceptual and empirical review. Clin Psychol: Sci Pract. (2003) 10:125. 10.1093/clipsy/bpg015

[2] Kabat-Zinn J. Mindfulness-based interventions in context: Past, present, and future. Clin Psychol: Sci Pract. (2003) 10:144–56. 10.1093/clipsy/bpg016

[3] Teasdale JD Moore RG Hayhurst H Pope M Williams S Segal ZV. Metacognitive awareness and prevention of relapse in depression: empirical evidence. J Consult Clin Psychol. (2002) 70:275–87. 10.1037/0022-006X.70.2.275 11952186

[4] Bishop S R Lau M Shapiro S Carlson L Anderson ND Carmody J. Mindfulness: a proposed operational definition. Clin Psychol: Sci Pract. (2004) 11:230–41. 10.1093/clipsy/bph077

[5] Tusaie K Edds K. Understanding and integrating mindfulness into psychiatric mental health nursing practice. Arch Psychiatr Nurs. (2009) 23:359–65. 10.1016/j.apnu.2008.10.006 19766927

[6] Brown KW Ryan RM. The benefits of being present: mindfulness and its role in psychological well-being. J Pers Soc Psychol. (2003) 84:822–48. 10.1037/0022-3514.84.4.822 12703651

[7] Davidson RJ. Empirical explorations of mindfulness: conceptual and methodological conundrums. Emotion. (2010) 10:8–11. 10.1037/a0018480 20141297 PMC4485421

[8] Creswell JD Myers HF Cole SW Irwin MR. Mindfulness meditation training effects on CD4+ T lymphocytes in HIV-1 infected adults: a small randomized controlled trial. Brain Behav Immun. (2009) 23:184–8. 10.1016/j.bbi.2008.07.004 18678242 PMC2725018

[9] Fang CY Reibel DK Longacre ML Rosenzweig S Campbell DE Douglas SD. Enhanced psychosocial well-being following participation in a mindfulness-based stress reduction program is associated with increased natural killer cell activity. J Altern Complem Med. (2010) 16:531–8. 10.1089/acm.2009.0018 20455784 PMC2921566

[10] Michalsen A Kunz N Jeitler M Brunnhuber S Meier L Lüdtke R. Effectiveness of focused meditation for patients with chronic low back pain-a randomized controlled clinical trial. Complement Ther Med. (2016) 26:79–84. 10.1016/j.ctim.2016.03.010 27261986

[11] Pathrose SP Everett B Patterson P Ussher J Salamonson Y McDonald F. Mindfulness-based interventions for young people with cancer: an integrative literature review. Cancer Nurs. (2021) 44:349–60. 10.1097/NCC.0000000000000821 32384422

[12] Park S Sato Y Takita Y Tamura N Ninomiya A Kosugi T. Mindfulness-based cognitive therapy for psychological distress, fear of cancer recurrence, fatigue, spiritual well-being, and quality of life in patients with breast cancer-a randomized controlled trial. J Pain Symptom Manage. (2020) 60:381–9. 10.1016/j.jpainsymman.2020.02.017 32105790

[13] Platt LM Whitburn AI Platt-Koch AG Koch RL. Nonpharmacological alternatives to benzodiazepine drugs for the treatment of anxiety in outpatient populations: a literature review. J Psychosoc Nurs Ment Health Serv. (2016) 54:35–42. 10.3928/02793695-20160725-07 27479478

[14] Yazdanimehr R Omidi A Sadat Z Akbari H. The effect of mindfulness-integrated cognitive behavior therapy on depression and anxiety among pregnant women: a randomized clinical trial. J Caring Sci. (2016) 5:195. 10.15171/jcs.2016.021 27752485 PMC5045953

[15] Sears SR Kraus S Carlough K Treat E. Perceived benefits and doubts of participants in a weekly meditation study. Mindfulness. (2011) 2:167–74. 10.1007/s12671-011-0055-4

[16] Shapiro SL Oman D Thoresen CE Plante TG Flinders T. Cultivating mindfulness: effects on well-being. J Clin Psychol. (2008) 64:840–62. 10.1002/jclp.20491 18484600

[17] Hofmann SG Gómez AF. Mindfulness-based interventions for anxiety and depression. Psychiatr Clin North Am. (2017) 40:739–49. 10.1016/j.psc.2017.08.008 29080597 PMC5679245

[18] Krygier JR Heathers JAJ Shahrestani S Abbott M Gross JJ Kemp AH. Mindfulness meditation, well-being, and heart rate variability: a preliminary investigation into the impact of intensive Vipassana meditation. Int J Psychophysiol. (2013) 89:305–13. 10.1016/j.ijpsycho.2013.06.017 23797150

[19] Dando LL Velitario AM Mallillin LLD Celdran MCB Alcantara JC. Mindful self-care and mental well-being of university health educators and professionals in Hail Region, Saudi Arabia. J Educ Health Promot. (2023) 12:351. 10.4103/jehp.jehp_1771_22 38144026 PMC10743852

[20] Taylor M Hageman JR Brown M. A mindfulness intervention for residents: relevance for pediatricians. Pediatr Ann. (2016) 45:e373–6. 10.3928/19382359-20160912-01 27735974

[21] Mackenzie CS Poulin PA Seidman-Carlson R. A brief mindfulness-based stress reduction intervention for nurses and nurse aides. Appl Nurs Res. (2016) 19:105–9. 10.1016/j.apnr.2005.08.002 16728295

[22] Sevel LS Finn MTM Smith RM Ryden AM McKernan LC. Self-compassion in mindfulness-based stress reduction: An examination of prediction and mediation of intervention effects. Stress Health. (2020) 36:88–96. 10.1002/smi.2917 31874122 PMC9281130

[23] Weinstein N Brown KW Ryan RM. A multi-method examination of the effects of mindfulness on stress attribution, coping and, emotional well-being. J Res Person. (2020) 43:374–385. 10.1016/j.jrp.2008.12.008

[24] Chen Y Yang X Wang L Zhang X. A randomized controlled trial of the effects of brief mindfulness meditation on anxiety symptoms and systolic blood pressure in Chinese nursing students. Nurse Educ Today. (2013) 33:1166–72. 10.1016/j.nedt.2012.11.014 23260618

[25] Heard PL Hartman S Bushardt SC. Rekindling the flame: using mindfulness to end nursing burnout. Nurs Manage. (2013) 44:24–9. 10.1097/01.NUMA.0000436366.99397.10 24157802

[26] Cohen-Katz J Wiley S Capuano T Baker DM Deitrick L Shapiro S. The effects of mindfulness-based stress reduction on nurse stress and burnout: a qualitative and quantitative study, part III. Holist Nurs Pract. (2005) 19:78–86. 10.1097/00004650-200503000-00009 15871591

[27] Cohen-Katz J Wiley SD Capuano T Baker DM Kimmel S Shapiro S. The effects of mindfulness-based stress reduction on nurse stress and burnout: a quantitative and qualitative study. Holist Nurs Pract. (2004) 18:302–8. 10.1097/00004650-200411000-00006 15624277

[28] Lan HK Subramanian P Rahmat N Kar PC. The effects of mindfulness training program on reducing stress and promoting well-being among nurses in critical care units. Austral J Adv Nurs. (2014) 31:22–31. 10.37464/2014.313.1594

[29] Gauthier T Meyer RML Grefe D Gold JI. An on-the-job mindfulness-based intervention for pediatric ICU nurses: a pilot. J Pediatr Nurs. (2015) 30:402–9. 10.1016/j.pedn.2014.10.005 25450445

[30] Gozalo RMG Tarrés JMF Ayora AA Herrero MA Kareaga AA Roca RF. Aplicación de un programa de mindfulness en profesionales de un servicio de medicina intensiva. Efecto sobre el burnout, la empatía y la autocompasión. Medicina intensiva. (2019) 43:207–16. 10.1016/j.medin.2018.02.005 29544729

[31] Hochschild A. Comment on Kemper's “social constructionist and positivist approaches to the sociology of emotions.” *Am J Sociol*. (1983) 89:432–4. 10.1086/227874

[32] Smith P. The Emotional Labor in Nursing. New York: Palgrave Macmillan. (1992).

[33] Grandey AA. Emotion regulation in the workplace: a new way to conceptualize emotional labor. J Occup Health Psychol. (2000) 5:95–110. 10.1037/1076-8998.5.1.95 10658889

[34] Diefendorff JM Croyle MH Gosserand RH. The dimensionality and antecedents of emotional labor strategies. J Vocat Behav. (2005) 66:339–57. 10.1016/j.jvb.2004.02.001

[35] Chien-Wen T. The important effect of employee's emotion management ability on his/her service behaviour in the international tourist hotel. Serv Ind J. (2009) 29:1437–49. 10.1080/02642060903026262

[36] Bodenheimer G Shuster S M. Emotional labour, teaching and burnout: investigating complex relationships. Educ Res. (2020) 62:63–76. 10.1080/00131881.2019.1705868

[37] Jeung DY Lee HO Chung WG Yoon JH Koh SB Back CY. Association of emotional labor, self-efficacy, and type a personality with burnout in korean dental hygienists. J Korean Med Sci. (2017) 32:1423–30. 10.3346/jkms.2017.32.9.1423 28776336 PMC5546960

[38] Isbell LM Tager J Beals K Liu G. Emotionally evocative patients in the emergency department: a mixed methods investigation of providers' reported emotions and implications for patient safety. BMJ Qual Saf. (2020) 29:1–2. 10.1136/bmjqs-2019-010110 31988259 PMC7382988

[39] Barnett MD Hays KN Cantu C. Compassion fatigue, emotional labor, and emotional display among hospice nurses. Death Stud. (2022) 46:290–6. 10.1080/07481187.2019.1699201 31814533

[40] Amiresmaili M Moosazadeh M. Determining job satisfaction of nurses working in hospitals of Iran: a systematic review and meta-analysis. Iran J Nurs Midwifery Res. (2013) 18:343–8. 24403934 PMC3877454

[41] Lee EK Ji EJ. The moderating role of leader-member exchange in the relationships between emotional labor and burnout in clinical nurses. Asian Nurs Res. (2018) 12:56–61. 10.1016/j.anr.2018.02.002 29463479

[42] Hwang WJ Park EH. Developing a structural equation model from Grandey's emotional regulation model to measure nurses' emotional labor, job satisfaction, and job performance. Appl Nurs Res. (2022) 64:151557. 10.1016/j.apnr.2021.151557 35307133

[43] Gulsen M Ozmen D. The relationship between emotional labour and job satisfaction in nursing. Int Nurs Rev. (2020) 67:145–54. 10.1111/inr.12559 31746014

[44] Delgado C Roche M Fethney J Foster K. Workplace resilience and emotional labour of Australian mental health nurses: results of a national survey. Int J Ment Health Nurs. (2022) 29:35–46. 10.1111/inm.12598 31050127

[45] Al-Hamdan ZM Muhsen A Alhamdan M Rayan A Banyhamdan K Bawadi H. Emotional intelligence and intent to stay among nurses employed in Jordanian hospitals. J Nurs Manag. (2020) 28:351–8. 10.1111/jonm.12932 31841256

[46] Fultz AR Walker M Lengerich A Bugajski A. Radiologic technologists' job satisfaction: a look at work environment, communication, and leadership. Radiol Technol. (2018) 89:536–40. 30420524

[47] Luksyte A Spitzmueller C Maynard DC. Why do overqualified incumbents deviate? Examining multiple mediators. J Occupat Health Psychol. (2011) 16:279–96. 10.1037/a0022709 21728436

[48] Kerstin A. Perceived overqualification and performance. The Role of the Peer-Group German. J Hum Resour Manag. (2013) 27:314–30. 10.1177/239700221302700403

[49] Erdogan B Bauer TN. Perceived overqualification and its outcomes: the moderating role of empowerment. J Appl Psychol. (2009) 94:57–565. 10.1037/a0013528 19271809

[50] Maynard DC Connelly CE Sauer CE. I'm too good for this job: Narcissism's role in the experience of overqualification. Appl Psychol. (2015) 64:208–32. 10.1111/apps.12031

[51] Van Dijk H Shantz A Alfes K. Welcome to the bright side: why, how, and when overqualification enhances performance. Hum Resour Manag Rev. (2020) 30:100688. 10.1016/j.hrmr.2019.04.004

[52] Khan J Ali A Saeed I Vega-Muñoz A Contreras-Barraza N. Person-job misfit: perceived overqualification and counterproctive work behavior. Front Psychol. (2020) 13:936900. 10.3389/fpsyg.2022.936900 35936320 PMC9355648

[53] Harari MB Manapragada A Viswesvaran C. Who thinks they're a big fish in a small pond and why does it matter? A meta-analysis of perceived overqualification. J Vocat Behav. (2017) 102:28–47. 10.1016/j.jvb.2017.06.002

[54] Chen S Cui H Zhou R Jia Y. Revision and reliability test of the masamune attention awareness scale. Chin J Clin Psychol. (2012) 20:148–51.

[55] Chun Y. Research on the impact of overqualification on employees' job shaping and job disengagement behavior. Technical report, Zhejiang University. (2014).

[56] Pinkawa C Dörfel D. Emotional labor as emotion regulation investigated with ecological momentary assessment- a scoping review. BMC Psychol. (2024) 12:69. 10.1186/s40359-023-01469-9 38347624 PMC10863272

[57] Botha E Gwin T Purpora C. The effectiveness of mindfulness based programs in reducing stress experienced by nurses in adult hospital settings: a systematic review of quantitative evidence protocol. JBI Evidence Synth. (2015) 13:21–29. 10.11124/jbisrir-2015-2380 26571279

[58] Bögels S Hoogstad B van Dun L de Schutter S Restifo K. Mindfulness training for adolescents with externalizing disorders and their parents. Behav Cogn Psychother. (2008) 6:193–209. 10.1017/S1352465808004190

[59] Lippold MA Duncan LG Coatsworth JD Nix RL Greenberg MT. Understanding how mindful parenting may be linked to mother-adolescent communication. J Youth Adolesc. (2015) 4:1663–73. 10.1007/s10964-015-0325-x 26162418 PMC4563867

[60] O'Day EB Butler RM Morrison AS Goldin PR Gross JJ Heimberg RG. Reductions in social anxiety during treatment predict lower levels of loneliness during follow-up among individuals with social anxiety disorder. J Anxiety Disord. (2021) 78:102362. 10.1016/j.janxdis.2021.102362 33486385 PMC7940567

[61] Wang Y Tang W Cao L Li Y. Self-concept clarity and Internet addiction disorder among junior high school students: a moderate mediation model. Front Psychiatry. (2022) 13:989128. 10.3389/fpsyt.2022.989128 36061278 PMC9433745

[62] Zhang CQ Chung PK Si G. Assessing acceptance in mindfulness with direct-worded items: the development and initial validation of the athlete mindfulness questionnaire. J Sport Health Sci. (2017) 6:311–20. 10.1016/j.jshs.2015.09.010 30356584 PMC6189008

[63] Keng SL Smoski MJ Robins CJ. Effects of mindfulness on psychological health: a review of empirical studies. Clin Psychol Rev. (2011) 31:1041–56. 10.1016/j.cpr.2011.04.006 21802619 PMC3679190

[64] Sohn BK Park SM Park IJ Hwang JY Choi J-S Lee Y-J. The relationship between emotional labor and job stress among hospital workers. J Korean Med Sci. (2018) 33:e246. 10.3346/jkms.2018.33.e246 30250411 PMC6146145

[65] Lu F Xu Y Yu Y Peng L Wu T Wang T. Moderating effect of mindfulness on the relationships between perceived stress and mental health outcomes among Chinese intensive care nurses. Front Psychiatry. (2019) 10:260. 10.3389/fpsyt.2019.00260 31057445 PMC6482227

[66] Kiken LG Shook NJ. Mindfulness and emotional distress: the role of negatively biased cognition. Person Indiv Differ. (2012) 52:329–333. 10.1016/j.paid.2011.10.031

[67] Wu W Lu Y. Is teachers' depression contagious to students? A study based on classes' hierarchical models. Front Public Health. (2022) 10:804546. 10.3389/fpubh.2022.804546 35387183 PMC8977487

[68] Wang IA Lin SY Chen YS Wu ST. The influences of abusive supervision on job satisfaction and mental health: the path through emotional labor. Personnel Rev. (2022) 51:823–38. 10.1108/PR-11-2018-0465

[69] Yin H. The effect of teachers' emotional labour on teaching satisfaction: moderation of emotional intelligence. Teach Teaching. (2015) 21:789–810. 10.1080/13540602.2014.995482

[70] Park HI O'Rourke E O'Brien KE. Extending conservation of resources theory: the interaction between emotional labor and interpersonal influence. Int J Stress Manag. (2014) 21:384–405. 10.1037/a0038109

[71] Scheepers RA Emke H Epstein RM Lombarts KM. The impact of mindfulness-based interventions on doctors' well-being and performance: a systematic review. Med Educ. (2020) 54:138–49. 10.1111/medu.14020 31868262 PMC7003865

[72] Oldershaw A Lavender T Sallis H Stahl D Schmidt U. Emotion generation and regulation in anorexia nervosa: a systematic review and meta-analysis of self-report data. Clin Psychol Rev. (2015) 39:83–95. 10.1016/j.cpr.2015.04.005 26043394

[73] Tremont G Davis JD Ott BR Galioto R Crook C Papandonatos GD. Randomized trial of the family intervention: telephone tracking-caregiver for dementia caregivers: use of community and healthcare resources. J Am Geriatr Soc. (2017) 65:924–30. 10.1111/jgs.14684 28008609 PMC5510656

[74] Incagli F Tarantino V Crescentini C Vallesi A. The effects of 8-week mindfulness-based stress reduction program on cognitive control: an EEG study. Mindfulness. (2019) 11:756–70. 10.1007/s12671-019-01288-3

[75] Kang D Yang JW Choi WJ Ham S Kang SK Lee W. Anxiety, depression and sleep disturbance among customer-facing workers. J Korean Med Sci. (2019) 34:e313. 10.3346/jkms.2019.34.e313 31833267 PMC6911873

[76] Meyer B Utter GL Hillman C. A personalized, interactive, cognitive behavioral therapy-based digital therapeutic (MODIA) for adjunctive treatment of opioid use disorder: development study. JMIR Mental Health. (2021) 8:e31173. 10.2196/31173 34623309 PMC8538017

[77] Maden-Eyiusta C Alten O. Expansion-oriented job crafting and employee performance:A self-empowerment perspective. Eur Manag J. (2020) 41:79–89. 10.1016/j.emj.2021.10.012

[78] Ma C Ganegoda D B Chen Z X Jiang X Dong C. Effects of perceived overqualification on career distress and career planning: mediating role of career identity and moderating role of leader humility. Hum Resour Manage. (2020) 59:521–36. 10.1002/hrm.22009

[79] Dong B Zheng B Wang Z. Career adaptability and turnover intention: a dual-mediation model. Career Dev Q. (2020) 68:145–57. 10.1002/cdq.12219

